# Cavernous lymphangioma of the urinary bladder in an adult woman: an additional case report of a rare lesion and literature review

**DOI:** 10.1186/s12894-021-00907-3

**Published:** 2021-10-13

**Authors:** Wei He, Li Jin, Fang-fang Lin, Xiao-long Qi, Xiang-lei He, Da-hong Zhang, Ming Zhao

**Affiliations:** 1 Department of Urology, Zhejiang Provincial People’s Hospital, People’s Hospital of Hangzhou Medical College, Hangzhou, 310014 Zhejiang China; 2 Department of Pathology, Laboratory Medicine Center, Zhejiang Provincial People’s Hospital, People’s Hospital of Hangzhou Medical College, Hangzhou, 310014 Zhejiang China; 3 Department of Pathology, Jiangshan People’s Hospital, Quzhou, 324100 Zhejiang China

**Keywords:** Lymphangioma, Bladder tumor, Mesenchymal tumor, Case report

## Abstract

**Background:**

Urinary bladder lymphangioma is a rare and benign lesion that is often causes symptoms related to irritation and urinary tract obstruction. Because a lymphangioma may resemble a true neoplasm of the urinary bladder clinically, the lesion must be removed for accurate histologic diagnosis and to rule out malignancy.

**Case presentation:**

We present a case of a 40-year-old female who was evaluated for painless gross hematuria. Clinical and diagnostic work up revealed a sharply defined mass involving the wall and bulging into the cavity on the dome of the bladder. Partial cystectomy was performed and histologic findings were compatible with cavernous lymphangioma. The symptom of hematuria relieved after the procedure and the patient was in good status without evidence of recurrence by cystoscopy at follow-up 6 months later.

**Conclusions:**

Lymphangioma of the urinary bladder is treated with surgical excision and seems to have no recurrence once completely resected, but long-time follow-up may be needed.

## Background

Lymphangioma is rare benign vascular lesion composed of a localized collection of dilated lymphatic channels, which has been classified into three histologic groups depending on the size of lymphatic spaces as capillary, cavernous, and cystic [1]. Lymphangioma usually affects children and infrequently occurs in adults, and is typically formed in the neck and axillary regions [2]. Lymphangioma is very rarely seen in the urinary bladder and to our knowledge only five such cases have been reported worldwide since 1983 [3–7]. This article describes an additional case of lymphangioma involving the urinary bladder in an adult Chinese woman and the clinical presentations, imaging characteristics as well as the gross and histologic features of the lesion are summarized.

## Case presentation

**Fig. 1:**
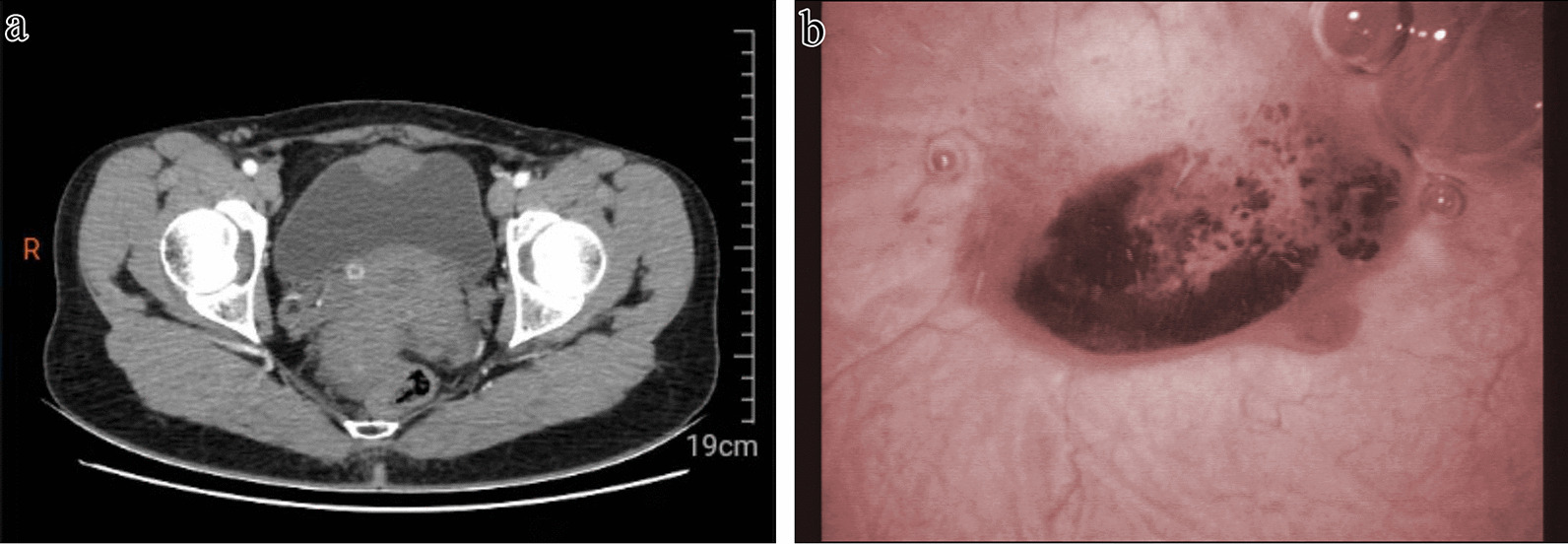
**a** Computerized tomography scan showed a sharply defined mass involving the dome of the urinary bladder wall. **b** Cystoscopy examination revealed a non-papillary tumor with a smooth surface with hemorrhage, bulging into the cavity on the dome of the bladder

A 40-year-old woman presented with painless gross terminal hematuria for 6 months. Urinalysis showed red blood cells notable for 65 per high power field and a negative urine culture. Computerized tomography (CT) scan showed a sharply defined, heterogeneously enhancing mass involving the dome of the urinary bladder wall measuring 4.0 cm in the maximum diameter (Fig. 1a). Cystoscopy examination revealed a red, non-papillary tumor with a smooth surface, bulging into the cavity on the dome of the bladder (Fig. 1b). Transurethral resection biopsy of the mass was carried out and histopathology revealed chronic inflammation with focally florid von Brunn's nests that cannot excluded a nested variant of urothelial carcinoma. A subsequent laparoscopically partial cystectomy (including the mass and a small amount of adjacent bladder tissues) was performed and the specimen was sent for intraoperative frozen section consultation, which indicated a benign mesenchymal lesion in favor of a hemangioma. The tumor was completely removed with negative resection margins. The symptom of hematuria relieved after the procedure and the patient was in good status without evidence of recurrence by cystoscopy at follow-up 6 months later.

**Fig. 2:**
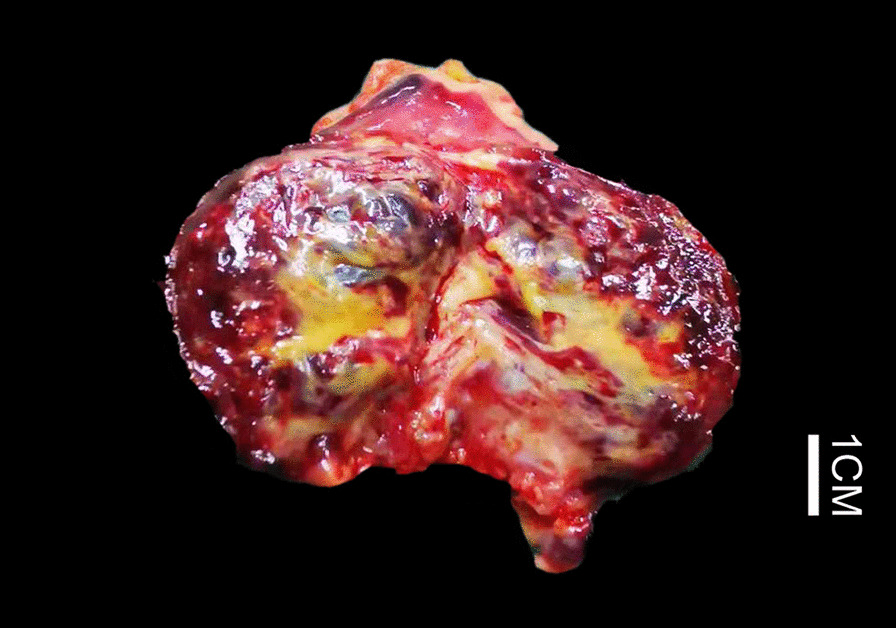
Gross examination demonstrated a predominantly solid tumor with numerous tiny dilated cysts containing chylous to yellow cheese-like fluid

**Fig. 3:**
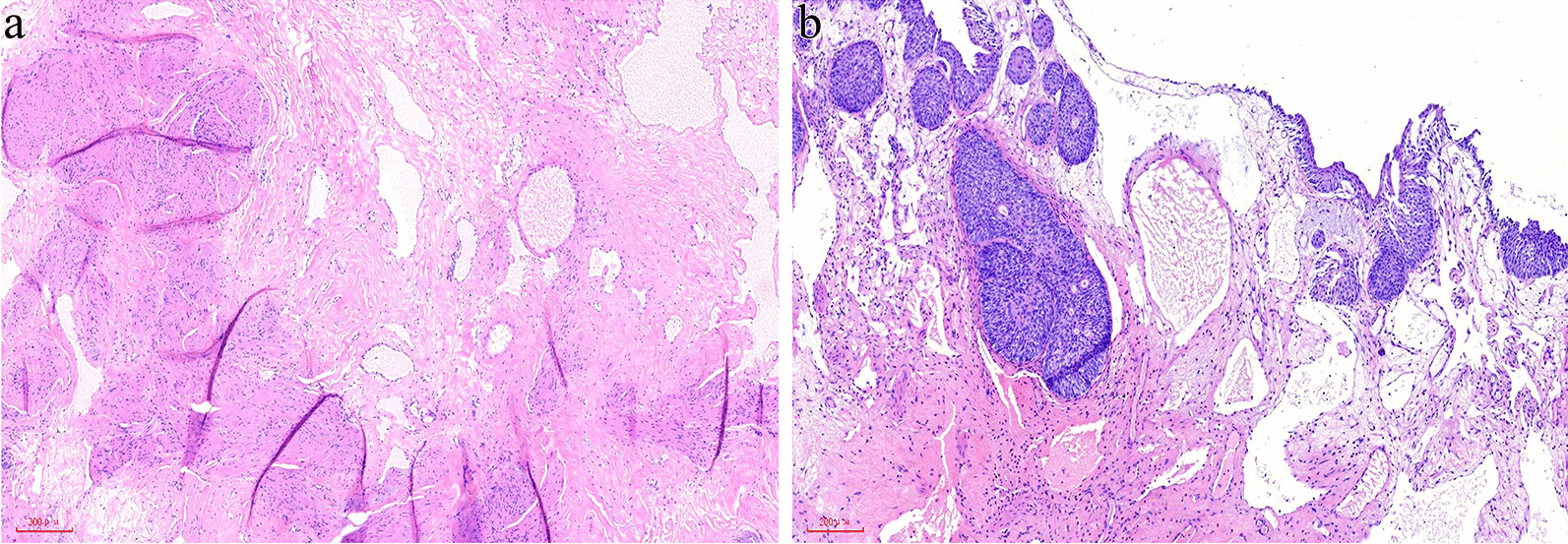
**a** Pathological findings revealed that the tumor was consisted of variably sized dilated lymphatic vessels which involved the full-thickness of the bladder wall and often dissected the muscularis propria. **b** In superficial mucosa there were clustering of thin-walled dilated lymphatics accompanied with focally florid von Brunn’s nests hyperplasia of the overlying urothelium

The cut surface of the resection specimen demonstrated a predominantly solid tumor of 3.8 cm in the maximum diameter and gray red in color with numerous tiny dilated cysts containing chylous to yellow cheese-like fluid (Fig. 2). The covered mucosa was unremarkable grossly. Microscopically, the tumor was consisted of variably sized dilated lymphatic vessels lined by flattened endothelium which involved the full-thickness of the bladder wall and often dissected the muscularis propria (Fig. 3a). In superficial mucosa the dilated lymphatics were thin-walled that connected with thick-walled often muscular, lymphatic channels in the deep muscularis propria, and the overlying urothelium showed flatten hyperplasia with focally florid von Brunn’s nests formation (Fig. 3b). By immunohistochemistry (IHC), the endothelium lining of the vascular channels showed positive stains for D2-40 and CD31, further confirming their lymphatic properties. Based the histologic and IHC features, the diagnosis of cavernous lymphangioma was rendered.

## Discussion and conclusions

Regarded as an abnormality of morphogenesis rather than as a neoplasm, lymphangioma is now also referred to as lymphatic malformation [2]. It affects almost any part of the body served by the lymphatic system but shows a predilection for the head, neck, and axilla and only sporadically occurs in internal organs or intra-abdominal locations where it can produce site-specific signs and symptoms [2].

Mesenchymal tumors of the urinary bladder are rare, and lymphangiomas among them are even rarer. Of the previously reported 5 patients with lymphangioma involving the urinary bladder (Table 1), 3 were adults and 2 were children, most of whom were manifested as painless gross hematuria, a symptom that is associated with hemorrhage of the lymphangioma or infection [5]. The first case reported by Bolkier et al. [3] in 1983 was a child with a 10 × 5 × 2 cm lesion starting from the wall of the bladder and invading the peritoneal cavity through the bladder wall. The second case reported by Wyle et al. [4] was a 49-year-old man presented with irritative voiding symptoms; cystoscopy showed a non-papillary tumor between the 2 orifices within the trigone. The third case reported by Niu et al. [5] was an 8-year-old girl whose tumor appeared as a shiny red mass in cystoscopy, and a bulge into the bladder on the right lateral wall was detected by imaging studies. The fourth case reported by Seyam et al. [6] was a 27-year-old woman; CT scan showed a heterogeneously enhancing mass arising from the anterior urinary bladder wall measuring 3.8 × 3.6 cm and cystoscopy showed a solid mass at the dome of the bladder covered by a normal mucosa. The fifth case reported by Moradi et al. [7] was a 40-year-old woman with a flat, 4 mm strawberry-like lesion on the right lateral wall of the bladder noticed by urethrocystoscopy.

**Table 1:** Clinical data from previous cases and the current case of lymphangioma of the urinary bladder

Case no./references	Age (years)	Sex	Clinical presentations	Cystoscopy features	Size (mm)	Location	Surgical approaches	Follow-up (months)
1/[3]	Child	NA	Painless macroscopic hematuria	NA	100	Lateral wall, unknown side	Partial cystectomy	NA
2/[4]	49	M	Irritative voiding symptoms	A non-papillary tumor with a smooth surface	NA	Between the two orifices within the trigone	Transurethral resection	Symptoms improved, 3
3/[5]	8	F	Terminal hematuria associated with intermitted fever over 1 week	A small red tumor bulging into the bladder cavity	5	Right lateral wall	Partial cystectomy	NED, 36
4/[6]	27	F	Painless gross hematuria for 6 months with occasional suprapubic pain	A mobile and solid mass covered by a normal mucosa	38	Dome	Robotic partial cystectomy	NED, 6
5/[7]	40	F	Alternative microscopic hematuria for 3 months	A flat highlighted strawberry-like lesion	4	Right lateral wall	Holmium laser ablation	NED, 24
6/current case	40	F	Painless gross hematuria for 6 months	A red, non-papillary tumor with a smooth surface, bulging into the cavity	38	Dome	Partial cystectomy	NED, 6

*F* female; *M* male; *NA* not available; *NED* no evidence of disease

Since its rarity, lymphangioma in the urinary bladder is extremely difficult to properly diagnose preoperatively, and histological examination is essential for the correct diagnosis. In most cases, the histologic diagnosis of lymphangioma is straightful, although some with secondary hemorrhage can resemble a cavernous hemangioma, and those with a significant amount of smooth muscle within the wall of the lymphatic vessels resemble a venous malformation. Histologic features that favor the diagnosis of lymphangioma over a hemangioma are lymphoid aggregates in the stroma and more irregular lumens with widely spaced nuclei [2]. IHC for lymphatic differentiation markers, such as D2-40, prospero homeobox 1(PROX1) and vascular endothelial growth factor receptor 3 (VEGFR3), is ultimately the most reliable means for distinguishing the two entities [8]. Similar to its cutaneous counterparts that often demonstrate hyperplasia of the epidermis [1], lymphangioma of the urinary bladder can also cause overlying urothelial hyperplasia and sometimes with florid von Brunn’s nests hyperplasia, which in cystoscopy biopsy may obscure the underlying lymphangioma and cause diagnostic confusions with urothelial carcinoma, especially for nested variant urothelial carcinoma.

Lymphangioma is typically benign, but because of its propensity for involvement of deeper tissue planes, recurrences have been documented in as many as 20% of patients after removal of superficial lesions [1]. Including our case, the 6 patients of bladder lymphangioma reported in the literature so far have not experienced tumor recurrence after surgery, but the follow-up time is limited (from 3 months to 3 years). Treatments consist of partial cystectomy for 4 patients, and transurethral resection and holmium laser ablation for one each patient [3–7]. Because urinary bladder lymphangioma is usually larger than it appears and often dissecting the full-thickness of the wall, complete resection is warranted to prevent recurrence. For this reason, partial cystectomy is the preferable treatment procedure than transurethral resection or laser ablation, particularly for lesions with larger size by cystoscopy or imaging evaluations.

In conclusion, lymphangioma arising in the urinary bladder is extremely rare, and only five cases have been identifed in studies reported in English. It is difficult to diagnose bladder lymphangioma according to its clinical features. The gold standard method used for its diagnosis is histopathology.

Lymphangioma of the urinary bladder is treated with surgical excision and seems to have no recurrence once completely resected, but long-time follow-up may be needed.

## Data Availability

Records and data pertaining to this case are in the patient’s secure medical records in Zhejiang Provincial People’s Hospital, People’s Hospital of Hangzhou Medical College. All searched data by literature review are included in this paper.

## Abbreviations

CT: Computerized tomography
IHC: Immunohistochemistry
PROX1: Prospero homeobox 1
VEGFR3: Vascular endothelial growth factor receptor 3
